# Endoscopic ultrasound-guided portal pressure gradient measurement: improving safety and overcoming technical difficulties

**DOI:** 10.1055/a-2109-0666

**Published:** 2023-07-13

**Authors:** Rafael Romero-Castro, Isabel Carmona-Soria, Victoria Alejandra Jiménez-García, Paula Fernández-Álvarez, Ángel Caunedo-Álvarez, Marc Giovannini, Atsushi Irisawa

**Affiliations:** 1 Division of Gastroenterology, Virgen Macarena University Hospital, Seville, Spain; 2 Digestive Unit, Vithas Hospital, Seville, Spain; 3 Endoscopic Unit, Paoli-Calmettes Institut, Marseille, France; 4 Dokkyo Medical University, Department of Gastroenterology, Shimotsuga, Tochigi, Japan

The hepatic venous pressure gradient obtained by interventional radiology is the current gold-standard, indirect method for quantifying the degree of portal hypertension 1 . Direct measurement of the portal pressure gradient (PPG) under endoscopic ultrasound (EUS) guidance using 25-gauge 2 3 and 22-gauge needles has been reported 4 .

We here report on EUS-guided PPG in 21 patients, with successful assessment in 19 (90 %) of these patients, using a dedicated 25-gauge needle (EchoTip Insight; Cook, Limerick, Ireland). Mean procedure time was 24 ± 12 minutes. In 4 patients anticoagulants were withdrawn before the procedure. One patient had transient epigastric pain 3 days after the procedure, which had been combined with bilobar liver biopsy; hospital admission was not required. No other adverse events were registered either immediately or 1 month later.

Technical difficulties encountered are demonstrated in Video 1 . In 2 cases (10 %), EUS-guided measurement of PPG failed because of exacerbated breathing movements and to unreliability of the pressure measurements, probably due to excessive bending of the echoendoscope and needle ( Fig. 1 ) and to use of the elevator and the up-and-down wheel. Thinner 25-gauge needles offer more flexibility and penetration ability than 22-gauge needles 5 . Occasionally, when puncturing the portal vein, even with a dedicated 25-gauge needle, the liver parenchyma is pushed away and the ultrasonographic window is momentarily lost. In such a case, the needle could puncture the hepatic artery. In 1 patient the 25-gauge needle passed close to the hepatic artery ( Fig. 2 ). We experienced difficulty in puncturing the wall of the hepatic vein in 1 case and the portal vein in 2 cases, having to traverse these vessels ( Fig. 3 , Fig. 4 ) and retrieve the needle.

**Video 1** EUS-guided PPG measurement: safety and technical aspects.


**Fig. 1:**
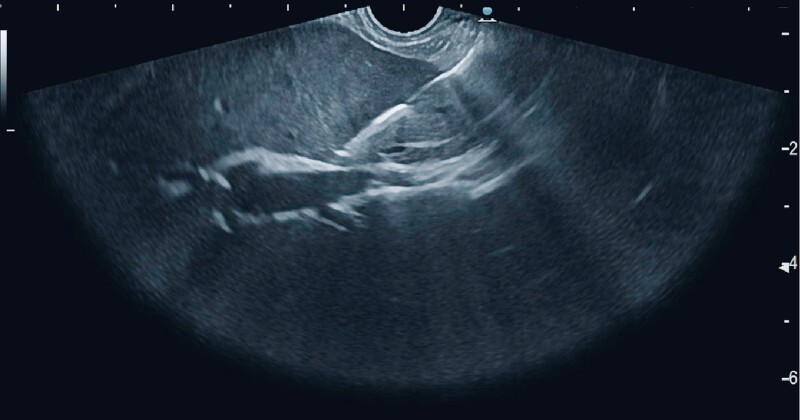
Bending of the needle displayed by endoscopic ultrasonography.

**Fig. 2:**
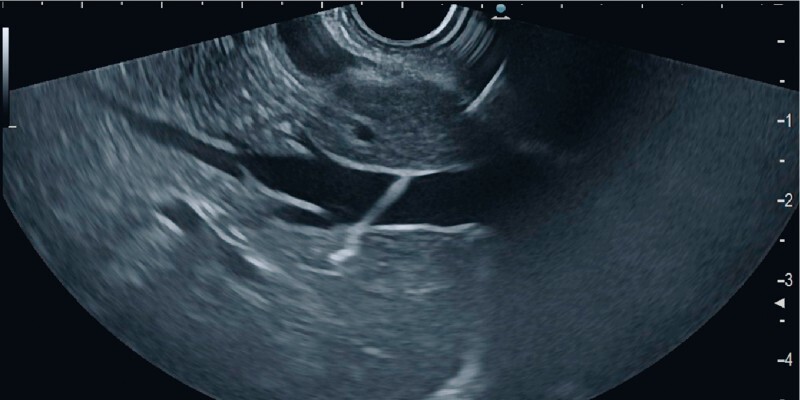
Endosonographic view of the dedicated 25-gauge needle traversing the hepatic vein.

**Fig. 3:**
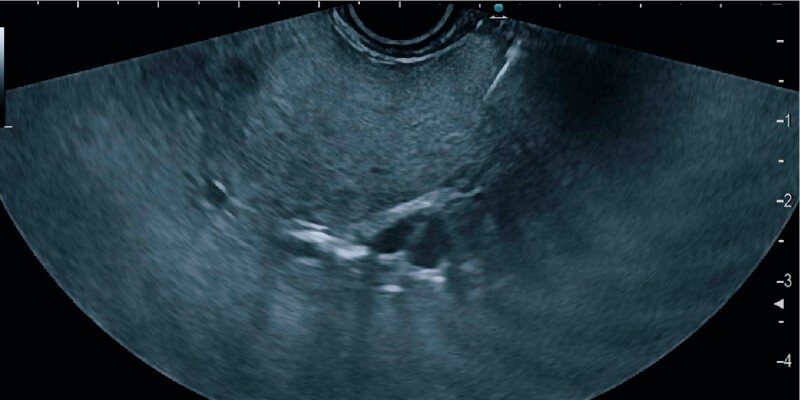
The left intrahepatic portal vein branch is traversed with a dedicated 25-gauge needle.

**Fig. 4:**
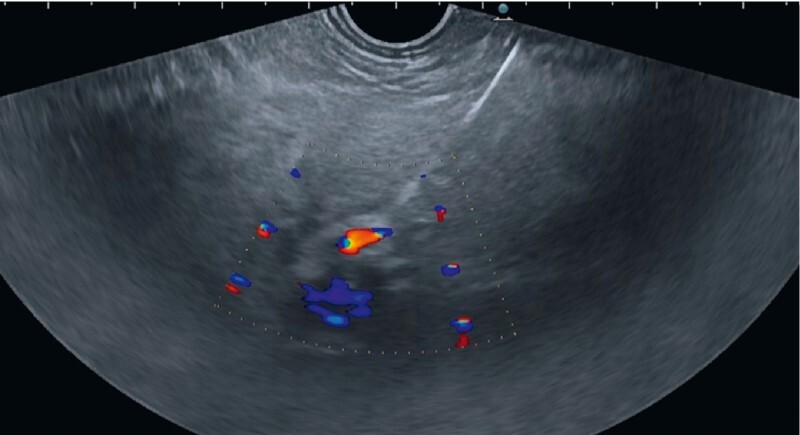
Endosonographic view of the dedicated 25-gauge needle inserted into the portal vein in very close proximity to the hepatic artery.

Table 1 shows the theoretical advantages of 25-gauge needles over 22-gauge needles in EUS-guided PPG measurement. To obtain reliable readings, forcing the elevator and the up-and-down wheel of the echoendoscope should be avoided.

**Table 1:** Possible pros and cons of dedicated 25-gauge needles vs. 22-gauge needles in endoscopic ultrasound-guided portal pressure gradient measurement

25-Gauge needles	22-Gauge needles
More flexibility and penetration ability	Less flexibility and penetration ability
Lower probability of adverse events	Higher probability of adverse events
Puncture of vessels easier	Puncture of vessels more cumbersome
Pressure measurement in narrow vessels more reliable	Pressure measure in narrow vessels less reliable with the needle in contact with the wall

In reporting our experience here, our aim is to help make the procedure of EUS-guided PPG measurement as safe and accurate as possible.

Endoscopy_UCTN_Code_TTT_1AS_2AG

## Notes

Endoscopy E-Videos

https://eref.thieme.de/e-videos
*E-Videos* is an open access online section of the journal *Endoscopy* , reporting on interesting cases and new techniques in gastroenterological endoscopy. All papers include a high-quality video and are published with a Creative Commons CC-BY license. Endoscopy E-Videos qualify for HINARI discounts and waivers and eligibility is automatically checked during the submission process. We grant 100% waivers to articles whose corresponding authors are based in Group A countries and 50% waivers to those who are based in Group B countries as classified by Research4Life (see: https://www.research4life.org/access/eligibility/ ). This section has its own submission website at https://mc.manuscriptcentral.com/e-videos
